# Supplementary material for: Polymer Modeling Reveals Interplay between Physical Properties of Chromosomal DNA and the Size and Distribution of Condensin-Based Chromatin Loops
Source: Genes (Basel). 2023 Dec 9;14(12):2193. doi: 10.3390/genes14122193 (PMC10742461; doi:10.3390/genes14122193)
Supplement: Supplementary file 1 [file genes-14-02193-s001.zip › (Genes) Supplementary Materials.pdf]

Supplementary materials

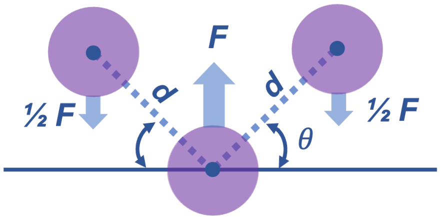

Figure S1. Persistence length in the model. Persistence length of a one-micron chain is set by varying its bending stiffness through a force that restores linear orientation of spring segments, also called hinge force. Hinge force is varied according to *hinge factor* (an input parameter). Hinge force is proportional to the angle of deviation from the horizontal between segments. At each time step in the model the restoring force acting on a bead is first obtained as described in our previous report. Then it is multiplied by empirically determined *hinge factor* and applied to the bead as well as the two conjoined beads to keep them colinear. For detailed description of restoring force see the bending rigidity section and Figure S3 in Lawrimore et.al. (2016) [17]

$$Position \begin{Bmatrix} x \\ y \\ z \end{Bmatrix}_i^{bead} (t) = F_i^{spring}(t) + F_i^{hinge}(t) + F_i^{random}(t) + F_i^{ExcludedVolume}(t) \quad \text{(S)}$$

Supplementary Equation of Motion. Position of every bead at simulation time (t) is governed by summation of four separate force terms. The spring force, F^{spring} is exerted on each bead via attached simple Newtonian springs. The hinge force, F^{hinge} restores linear orientation and is specific to bending stiffness parameter of the chain (see above). Random fluctuation of beads due to temperature (Brownian motion, k_BT), cellular ATP, and opposed by viscous drag is captured by F^{random} term. The excluded volume term, $F^{ExcludedVolume}$ is necessary to model the self-avoiding behavior of our worm-like chains.

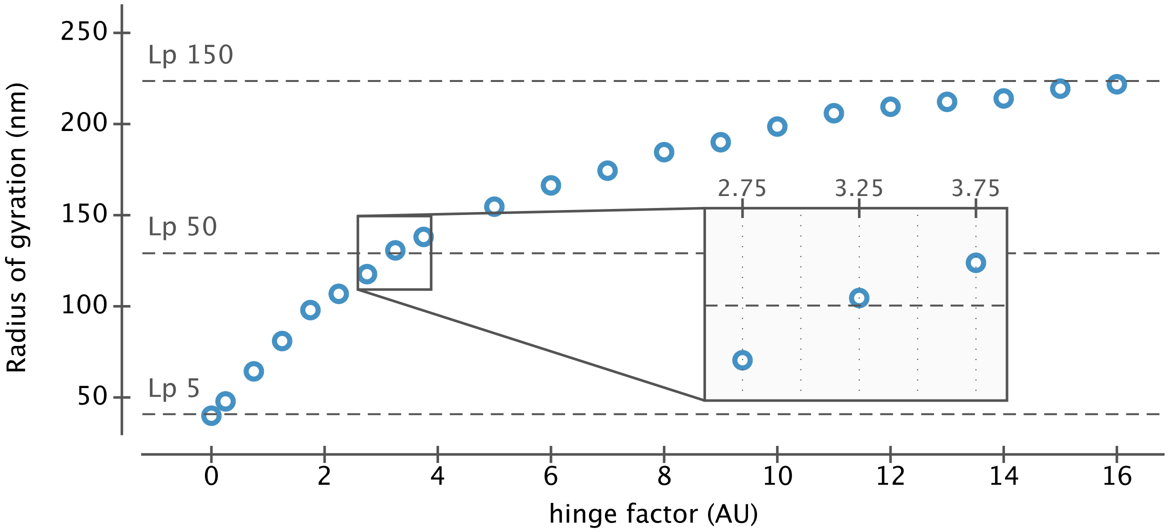

Figure S2. Average Radii of gyration (R_g) of one-micron chain at observable plateau as a function of *hinge factor*. Each point is an average R_g of collapsed chain obtained from N = 8 independent simulations. Dotted lines show expected theoretical R_g for one-micron chains of given stiffness: L_P = 5 nm (100 segments), L_P = 50 nm (10 segments), L_P = 150 nm (3.3333 segments). To set a corresponding L_P in our model we choose empirically determined *hinge factor* for which the R_g of simulated chain is closest to the theoretical values shown by dotted lines. We choose three *hinge factors* to model chains as Floppy = 0; Stiff = 3.25, and Very stiff = 16. See Table 1 in main text for summary of values used.

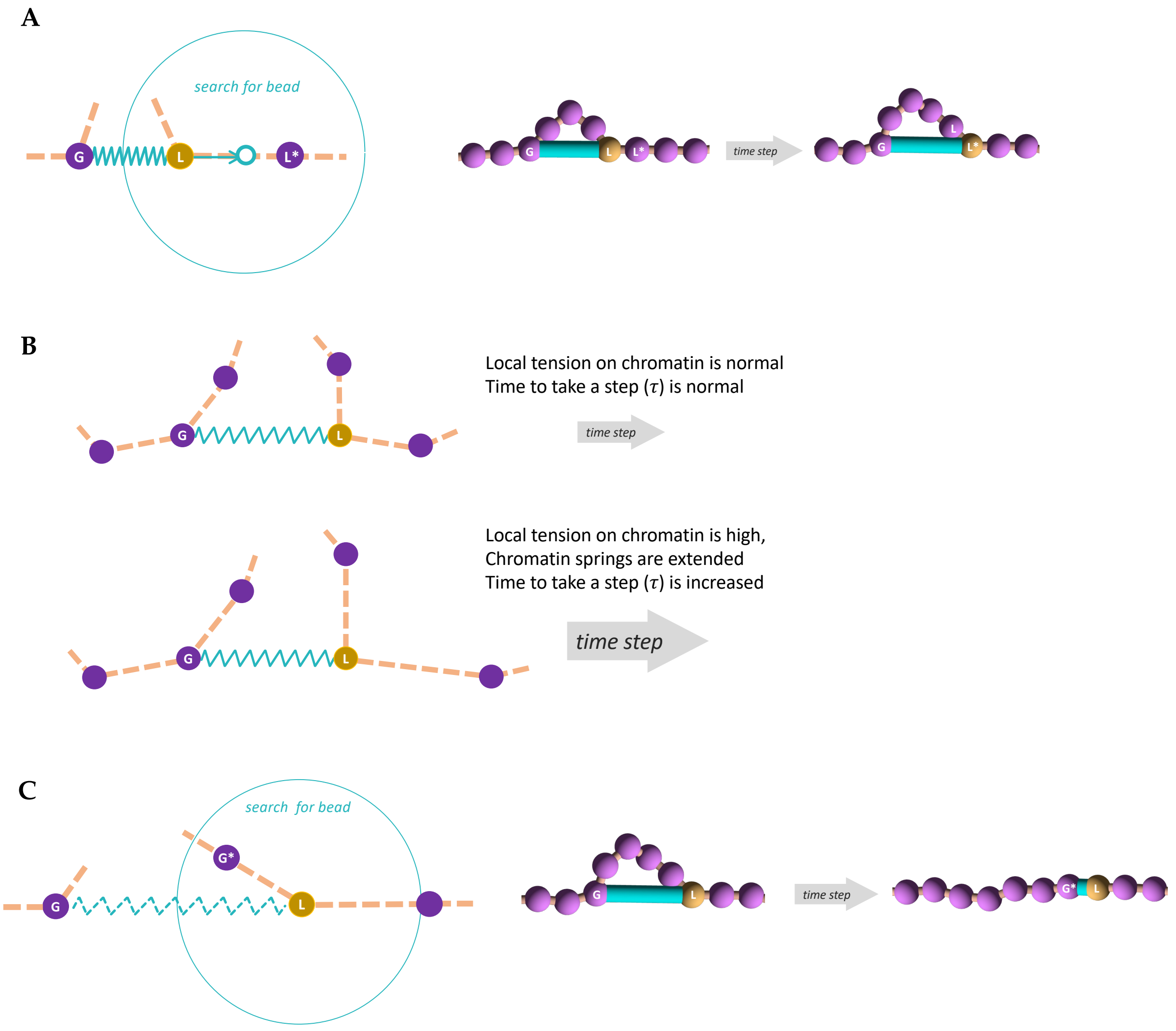

Figure S3. Condensin crosslinking spring. (**A**) Modelled as a dynamic spring crosslinking two beads condensin takes a step in the direction away from lagging bead (G). When a nearest bead is found it becomes new leading bead (L*). The spring unbinds existing leading bead (L) and binds new bead (L*). In example above the loop size is increased by one bead. (**B**) Stepping time is increased when tension on local chromatin is high. Increased lengths of local chromatin springs delay extrusion step consequently throttling (or stalling) condensin extrusion. (**C**) Condensin spring under high tension can extend up to 30 nm (critical length). Above 30 nm condensin spring releases its lagging bead (G), searches for a bead in proximity of the leading bead (L). Nearest bead found (G*) becomes the lagging bead and a new spring will crosslink it to the leading bead (L). The search for nearest bead is agnostic to ordinal bead numbers in the chain. The nearest bead can be anywhere on the chain, and a reversal of stepping direction is possible. In example (C) above the new lagging bead (G*) is immediately behind and next to the leading bead (L), such that a single step destroys existing loop and stepping direction is maintained. On extended chains a directional traversal of the chain by condensins is frequently observed (See Supplementary Movie SM3). Select visualizations of condensin spring stepping on chains can be found in Supplementary Movies SM4-SM6.

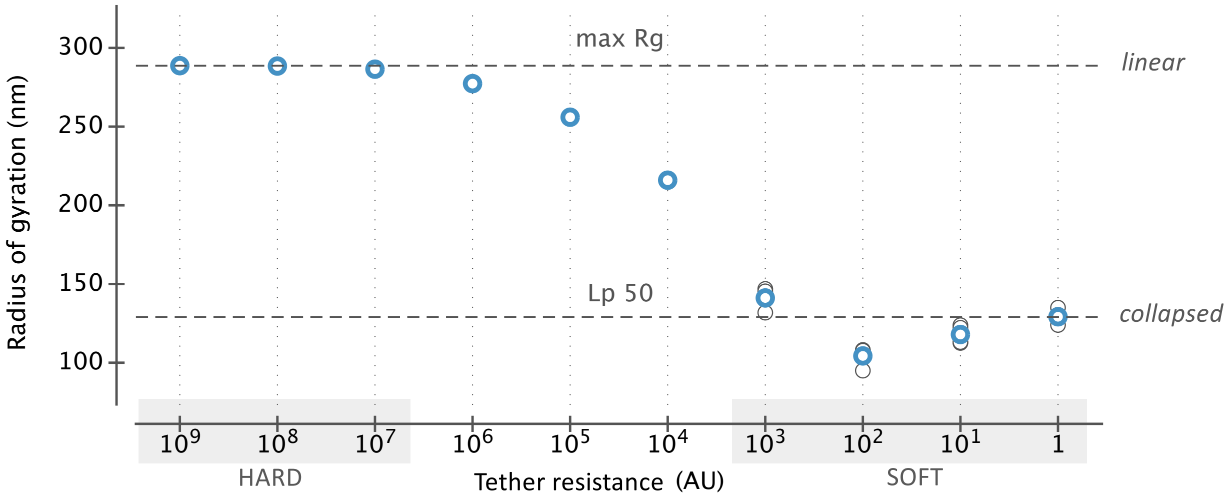

Figure S4. Relationship between R_g of a collapsed chain and tether resistance of its ends. Reducing tether resistance on the end beads recapitulates a change in chain's R_g ranging from anchored (fully constrained) to unconstrained (free ends) chains. Blue circles are average radii of gyration at observable plateau from $N = 4$ independent simulations over simulation time (35 ms). Dotted lines show theoretical R_g (collapsed) for one-micron L_p = 50 nm chain and computed maximum R_g (linear) of a fully extended one-micron chain.

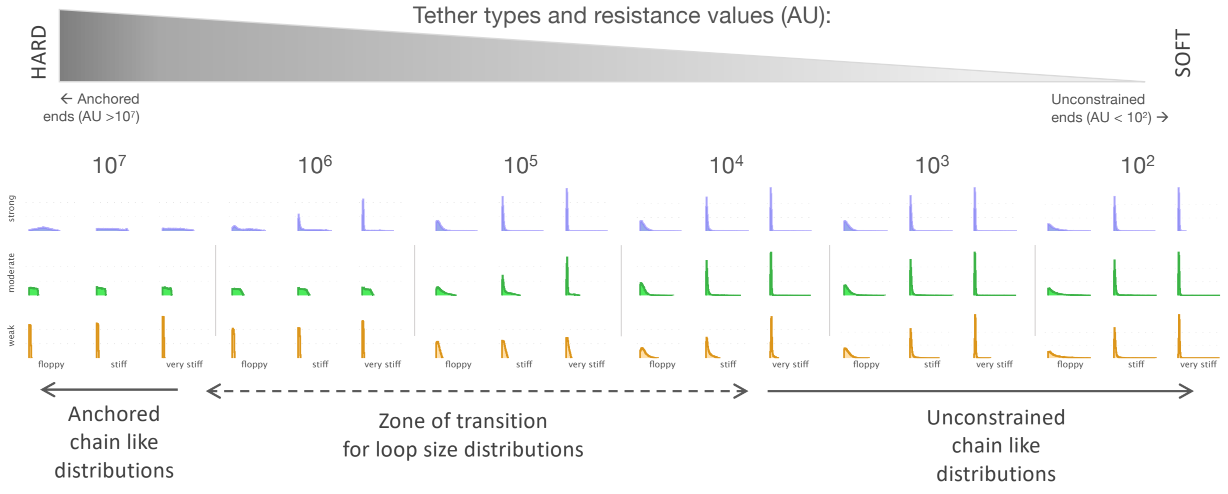

Figure S5. Histograms of loop size distributions as tethers change from hard to soft. The range modeled is from 10^7 AU to 10^2 AU tether resistance on the end beads.

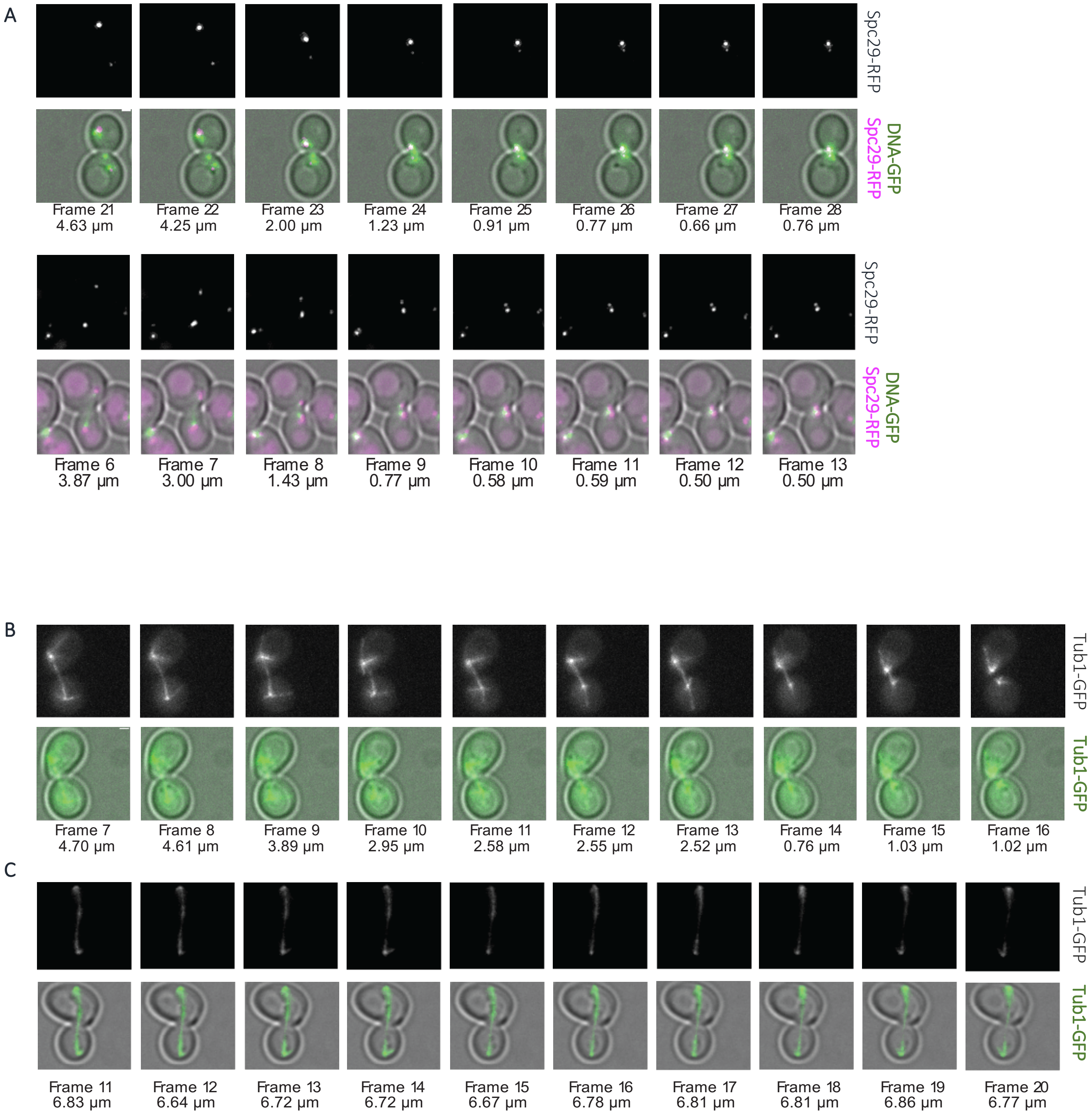

Figure S6. Example images of SPB-SPB recoil (with snapback events) measured in dicentric KBY6201a and a spindle collapse in dicentric KBY7004 (with and without snapback). (**A**) Two examples of KBY6201a cells with SPB-SPB recoil in anaphase. Spindle pole body (Spc29-RFP) in red channel (top panels) and overlapped with DNA in green (LacO::lacI-GFP) in composite images (bottom panels). (**B**) Snapback event and (**C**) no snapback event in KBY7004 with Tubulin-GFP visualized in single channel and in composite images.
